# Positive Crosstalk of MAMP Signaling Pathways in Rice Cells

**DOI:** 10.1371/journal.pone.0051953

**Published:** 2012-12-14

**Authors:** Yoshitake Desaki, Ippei Otomo, Daijiro Kobayashi, Yusuke Jikumaru, Yuji Kamiya, Balakrishnan Venkatesh, Shinji Tsuyumu, Hanae Kaku, Naoto Shibuya

**Affiliations:** 1 Department of Life Sciences, School of Agriculture, Meiji University, Kawasaki, Kanagawa, Japan; 2 RIKEN Plant Science Center, Yokohama, Kanagawa, Japan; 3 Faculty of Agriculture, Shizuoka University, Shizuoka, Japan; University of Hyderabad, India

## Abstract

Plants have evolved efficient defense mechanisms known as priming and synergy, both of which can mobilize defense responses more extensively against successive pathogen invasion or simultaneous stimulation by different signal molecules. However, the mechanisms underlying these phenomena were largely unknown. In the present study, we used cultured rice cells and combination of purified MAMP molecules as a model system to study the mechanisms of these phenomena. We found that the pretreatment of rice cells with a low concentration of bacterial lipopolysaccharide (LPS) apparently primed the defense responses induced by successive *N*-acetylchitooctaose (GN8) treatment. On the other hand, simultaneous treatment with GN8 and LPS also resulted in the similar enhancement of defense responses observed for the LPS-induced priming, indicating that the synergistic effects of these MAMPs are basically responsible for such enhancement of defense responses, though the effect could be interpreted as “priming” under some experimental conditions. These results also indicate that such a positive crosstalk of signaling cascade downstream of MAMP receptors seems to occur very rapidly, probably at early step(s) of signaling pathway. Comprehensive analysis of phytohormones revealed a specific enhancement of the synthesis of jasmonic acid (JA), both in the LPS pretreatment and also simultaneous treatment, indicating a role of JA in the enhancement of downstream responses.

## Introduction

Plants have the ability to initiate various defense responses through the perception of microbe-associated molecular patterns (MAMPs) by corresponding pattern recognition receptors. MAMPs are the conserved and indispensable microbe specific molecules such as fungal cell wall polysaccharides (chitin and β-glucan), bacterial flagellin, peptidoglycan, elongation factor Tu [1]–[3]. MAMP-induced defense responses are very important as the first layer resistance in plant immunity [4], [5]. It has also been known that damage-associated molecular patterns (DAMPs), such as oligogalacturonides, induce defense responses in plants.

Lipopolysaccharide (LPS) is a typical component of the cell walls of gram-negative bacteria and has been known to induce defense responses in various dicot plants [6]. We also reported that LPS induces defense responses associated with programmed cell death (PCD) in cultured rice cells [7]. On the other hand, several papers reported that LPS has an ability to establish primed state in plants [8], a phenomenon in which plants can more rapidly and extensively mount defense responses to successive pathogen invasion [9]. In the case of priming, LPS does not induce defense responses directly, but idles defense machinery for a quicker response to successive invasion. Priming has been described to occur during the activation of systemic acquired resistance (SAR) and induced systemic resistance (ISR) [10], [11]. Signaling cascade leading to the primed state has been proposed to involve phytohormones such as salicylic acid (SA) and jasmonic acid [10], [12]–[14]. These phytohormones and their analogs, such as benzothiadiazol (BTH), have been reported to induce primed state in plants [15]. Manipulation of this ability of plants to establish a novel disease resistant crop seems to be advantageous compared to the strategy that includes the constitutive activation of defense machinery, because the former approach could minimize the unfavorable effects on the growth and quality of agricultural products caused by the useless activation of defense responses [16]. Concerning to the mechanism of priming, it has been reported that the accumulation of inactive form of signaling components such as mitogen activated kinases (MAPKs) and transcription factors, or their mRNAs contributes for the establishment of primed state in SAR [17]–[19]. However, detailed analysis of molecular mechanisms leading to the establishment of primed state still remains to be elucidated.

On the other hand, plants also have another way to activate defense system effectively, so-called synergy, which has been known as synergistic activation of defense responses by simultaneously added multiple stimuli. It has been reported that simultaneous recognition of multiple MAMP/DAMP molecules could induce more rapid and extensive defense responses than the one expected from the simple addition of the responses induced by each MAMP/DAMP molecule, indicating the presence of synergistic interaction between the signaling pathways downstream of the corresponding receptors [20], [21]. The presence of such a system seems to reflect the situation plants encounter under natural conditions. Even a single bacterium or fungus carries different types of MAMPs, that is, flagellin, LPS, peptidoglycan and EF-Tu as known MAMPs for bacteria and chitin and β-glucan for fungi. It is also possible that a plant is exposed to simultaneous invasion by multiple pathogenic microbes. Furthermore, invasion of pathogenic microbes is often associated with the damage of infected tissue, generating some types of DAMP molecules. Thus, if simultaneous perception of different MAMP/DAMP molecules facilitates effective activation of defense machinery, it is advantageous for plants. However, the molecular mechanism leading to such a synergistic activation of defense responses is not clear.

Both priming and synergy seem to be the phenomena reflecting some types of positive crosstalk between defense signaling pathways and could be utilized to establish disease resistant plants or develop agrochemicals in a unique way. A major difficulty to study detailed mechanisms of these phenomena, which is prerequisite for such applications, lies in the limited availability of purified MAMP/DAMP molecules and also a suitable model system for such a study. In this paper, we analyzed the priming effect of LPS preparations from several bacterial strains on chitin oligosaccharide-induced defense responses in cultured rice cells. We found that the pretreatment of rice cells with a low concentration of LPS, which did not induce detectable defense responses by itself, clearly primed the defense responses, such as ROS generation and defense gene expression, induced by successive chitin oligosaccharide treatment. On the other hand, simultaneous treatment with chitin oligosaccharide and LPS resulted in the similar enhancement of defense responses observed for the LPS-induced priming, indicating that the synergistic effects of these MAMPs are basically responsible for such enhancement of defense responses, though the effects could be interpreted as “priming” under some experimental conditions. These results also indicated that the positive crosstalk of signaling cascade downstream of MAMP receptors seems to occur very rapidly, probably at early step(s) of signaling pathway. Comprehensive analysis of phytohormones revealed a specific enhancement of the synthesis of jasmonic acid (JA), both in the LPS pretreatment and also simultaneous treatment, indicating a role of JA in the enhancement of downstream responses.

## Results

### LPS Pretreatment Establishes Primed State in Rice Cells for Chitin-induced Defense Responses

**Figure 1:**
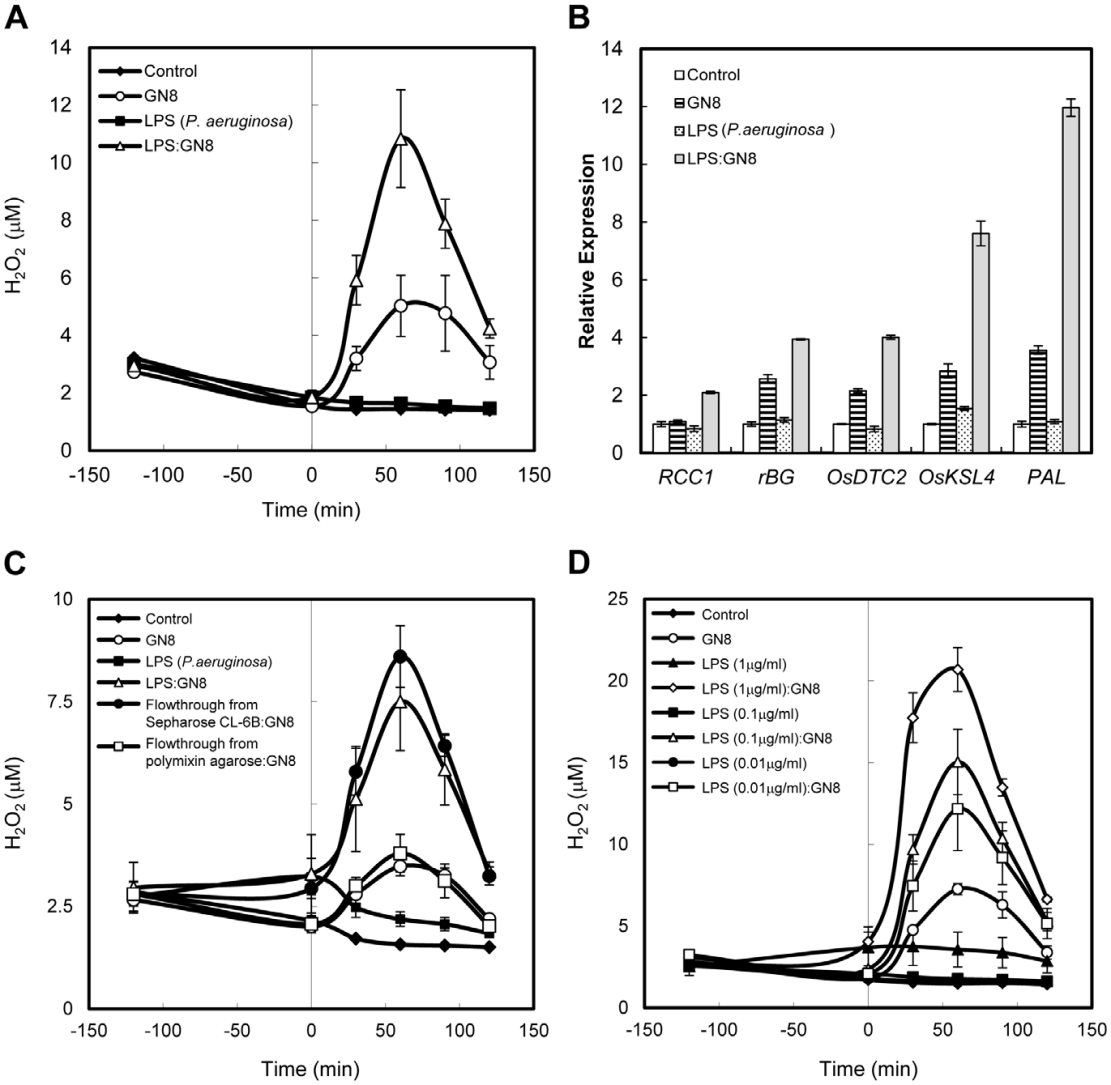
LPS pretreatment primed chitin-induced defense responses in rice cells. (A) Priming of GN8-induced ROS generation by the pretreatment with *P. aeruginosa* LPS. (B) Priming of GN8-induced expression of defense related genes by the pretreatment with *P. aeruginosa* LPS. Relative expression to the water control was shown. *RCC1* (AK061042), rice class 1 chitinase; *rBG* (AK058891), rice β 1,3-glucanase; *OsDTC2* (AK108710), stemar-13-ene synthase; *OsKSL4* (AK119327), 9βH-pimara-7,15-diene synthase; *PAL* (AK068993), phenylalanine anmonia lyase. (C) Confirmation of LPS as the active component for priming activity. Commercial *P. aeruginosa* LPS preparation was applied to either a polymixin B-agarose column or a sepharose CL-6B column, and eluted with distilled water. Priming activity in the commercial LPS preparation was completely recovered in the flow-through fraction from the sepharose CL-6B column. On the other hand, the flow-through fraction from polymixin B-agarose column did not show detectable priming activity. (D) Dose dependency of priming activity of *P. aeruginosa* LPS on GN8-induced ROS production. LPS concentration used for the experiments (A) to (C) was 0.1 µg/ml and GN8 concentration for the experiments (A) to (D) was 0.08 ng/ml. Rice cells were pretreated with the LPS for 120 min at 25°C and successively treated with GN8. Error bars indicate standard deviation.

Successive treatment with two MAMP molecules, Lipopolysaccharide (LPS) and *N*-acetylchitooctaose (GN8), in suspension-cultured rice cells was used as a model system to evaluate the priming activity of LPS. The expression of defense-related genes for PR proteins (chitinase and β-glucanse) and enzymes for phytoalexin biosynthesis (*PAL*, *OsDTC2*, *OsKSL4*) and also ROS generation, which has been reported to be generated mainly by cell surface NADPH oxidase [22], [23], were used as the index of defense responses. In this paper, we applied the term “priming” if a defense-related cellular response induced by an elicitor is enhanced by some pretreatment (for example, LPS pretreatment) than a simple sum of the cellular response induced by each treatment. By using this system, we found that the pretreatment of rice cells with a low concentration of LPS preparation from *Pseudomonas aeruginosa* (0.1 µg/ml), which did not induce detectable defense responses at this concentration, clearly enhanced the induction of ROS generation and defense gene expression by successive GN8 treatment (0.08 ng/ml = 50 pM) (Fig. 1A, B). This priming activity for successive GN8 treatment was not only observed for *P. aeruginosa* LPS, but also observed for LPS preparations obtained from 3 other bacterial species including 2 phytopathogens (Fig. S1), indicating that many bacterial LPSs have the capacity to prime rice cells for successive elicitor treatment.

Although we used LPS preparations purified from pathogenic bacteria by ourselves and also commercial preparations with the highest purity, we still anticipated the possibility of contamination of other bacterial components. To exclude such a possibility, we utilized specific adsorption of LPS on polymyxin B-agarose, which binds LPS through the interaction with Lipid A portion, thus enabling specific removal of LPS from other bacterial components [7]. When the commercial LPS preparation from *P. aeruginosa* was applied to the polymyxin B-agarose column, the flow-through fraction did not show detectable priming activity (Fig. 1C). On the other hand, when the same LPS preparation was applied to a control agarose (Sepharose CL-6B) column without fixed polymyxin B, LPS was not trapped and the flow-through fraction showed clear priming activity. These results confirmed that the priming activity detected in the commercial LPS preparations resides in the LPS molecules.

Analysis of dose-dependency of *P. aeruginosa* LPS for the priming activity showed that it could enhance ROS generation induced by the successive GN8 treatment even at a dosage of <0.01 µg/ml, which was much lower than the minimal concentration with which detectable defense responses were induced by the LPS alone (Fig. 1D).

### Factors Potentially Relating to the LPS Mediated Priming

**Figure 2:**
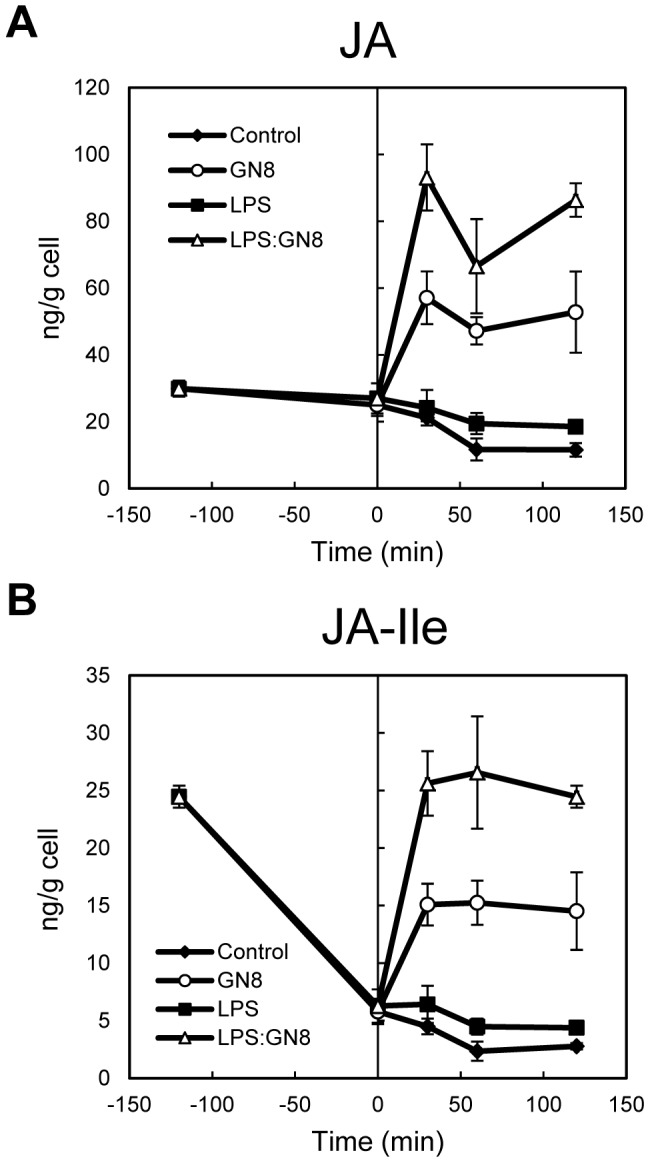
Cellular responses primed by LPS pretreatment. (A, B) Changes in JA and JA-Ile concentrations during LPS pretreatment and after successive GN8 treatment. Both GN8-induced accumulation of JA and JA-Ile were clearly primed by LPS pretreatment, though the concentrations of these compounds did not change during the pretreatment. Experimental conditions were the same to Fig. 1.

Phytohormones such as JA and SA have been suggested to play a role in the establishment of primed state in plants [10], [12], [14]. To evaluate the contribution of phytohormones for the LPS-mediated priming, we performed a comprehensive analysis of phytohormones (JA, JA-Ile, SA, ABA, IAA, GAs, CKs) after LPS pretreatment. No change in the concentration of these phytohormones was observed after LPS pretreatment (0.1 µg/ml) (Fig. 2A, B and Fig. S2). On the other hand, accumulation of JA and its bioactive form, JA-Ile, after successive GN8 treatment (0.08 ng/ml) was clearly enhanced (Fig. 2A, B), indicating the possible involvement of these compounds in the priming of downstream responses such as rice phytoalexin biosynthesis [24]. Although the level of JA-Ile significantly decreased during the pretreatment, the concentration at the start of GN8 addition was almost the same for all treatments. No other phytohormone showed similar enhancement of accumulation.

### Simultaneous Treatment with LPS and other MAMPs Synergistically Activate Defense Responses

**Figure 3:**
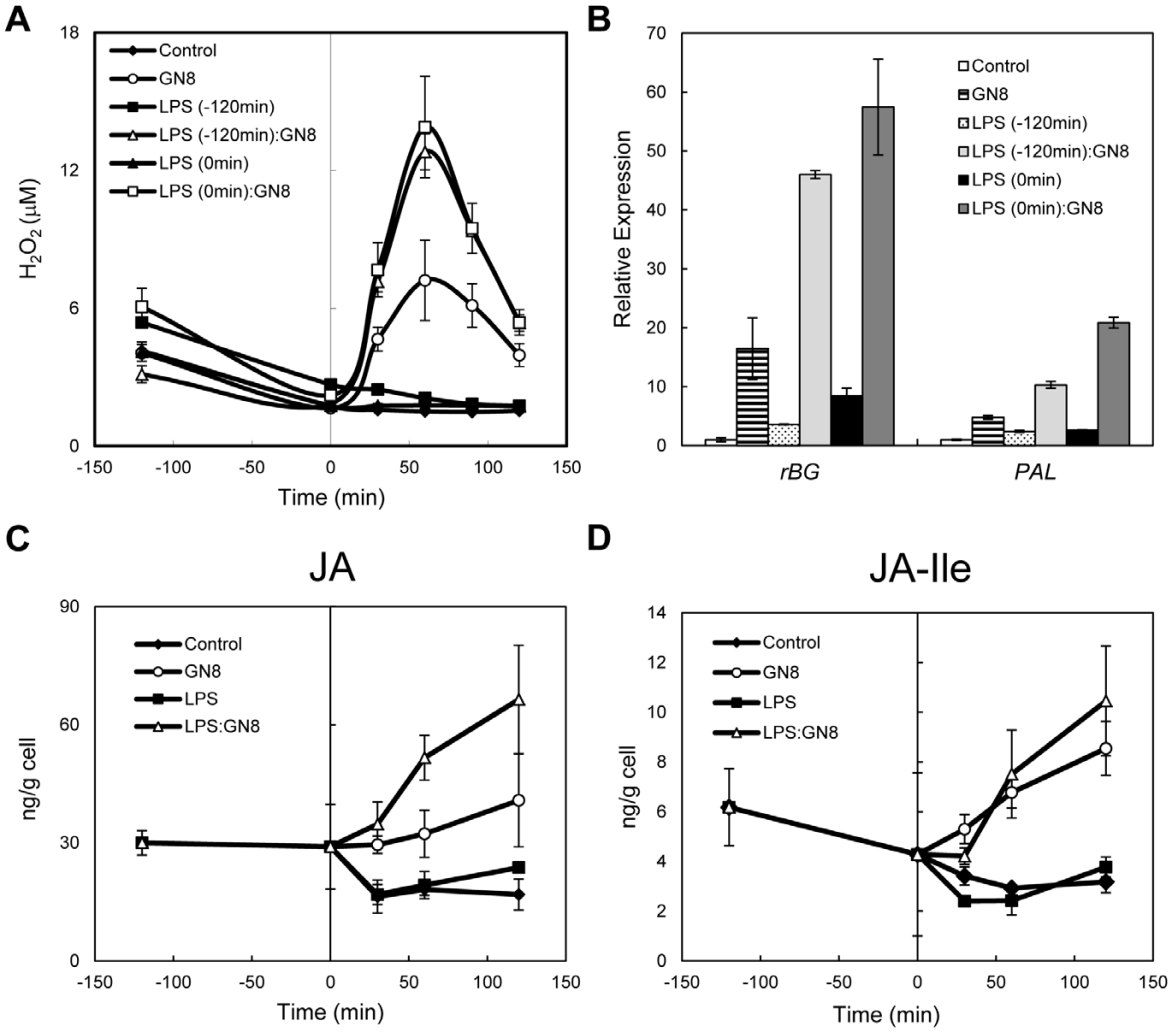
Comparison of “priming” and “synergy”. (A, B) ROS generation and defense gene expression induced by simultaneous treatment with LPS and GN8, or LPS pretreatment (-120 min) and successive GN8 treatment (0 min). LPS concentration was 0.1 µg/ml and GN8 concentration was 0.08 ng/ml. (C, D) Changes in JA and JA-Ile concentration after simultaneous treatment with LPS (0.1 µg/ml) and GN8 (0.08 ng/ml). Synergistic effect was observed for the accumulation of JA but not for JA-Ile. Experimental conditions were the same to Fig. 1.

Based on the observations described so far, we were interested to examine the time length required for the establishment of primed state by LPS pretreatment. Interestingly, when we changed the time of LPS pretreatment from 120 min to 0 min ( = simultaneous treatment), we found there is no difference between the enhancement of GN8-induced ROS generation in both treatment (LPS, 0.1 µg/ml; GN8, 0.08 ng/ml) (Fig. 3A), indicating that at least the enhancement of such early responses could be interpreted as a synergistic effect rather than priming. Synergistic effect was also observed for the expression of several defense-related genes (Fig. 3B) and the accumulation of JA, though the significant enhancement of JA-Ile was not observed in this case (Fig. 3C, D and Fig. S3).

**Figure 4:**
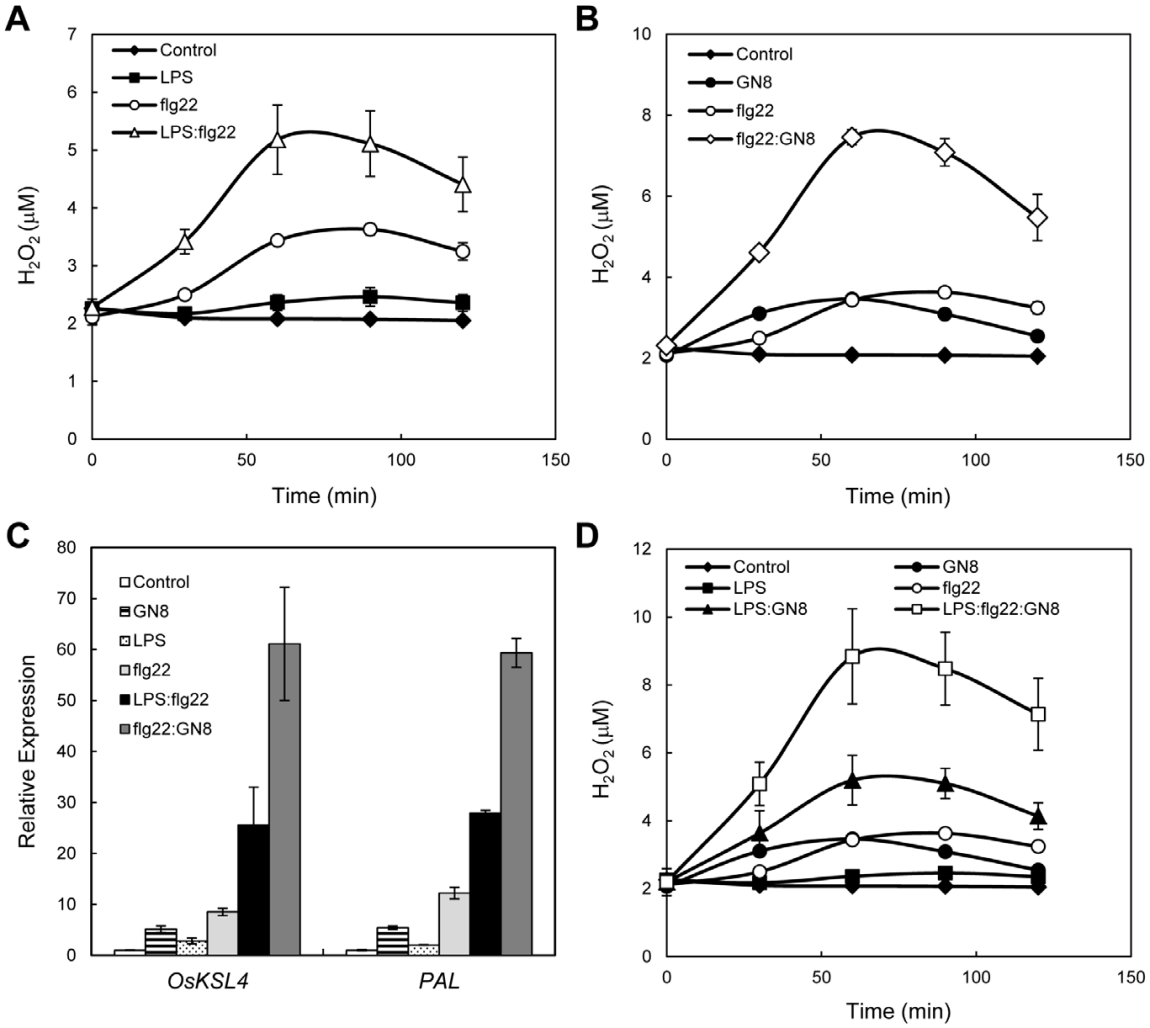
Synergistic enhancement of defense responses by different combination of MAMPs. (A) Synergistic induction of ROS generation by simultaneous treatment of LPS (0.1 µg/ml) and flg22 (1 ng/ml). (B) Synergistic induction of ROS generation by simultaneous treatment of GN8 (0.08 ng/ml) and flg22 (1 ng/ml). (C) Synergistic induction of defense gene expression by LPS:flg22 or GN8:flg22. (D) Comparison of ROS generation induced by simultaneous treatment with two or three MAMP elicitors. Experimental conditions were the same to Fig. 1.

Synergistic enhancement of defense responses was not only observed for the combination of LPS and GN8 but also observed for different sets of MAMP elicitors, i.e., LPS/flg22 and GN8/flg22 (LPS, 0.1 µg/ml; GN8, 0.08 ng/ml; flg22, 1 ng/ml = 0.44 nM) (Fig. 4A, B, C). Moreover, simultaneous treatment with LPS, GN8 and flg22 showed more enhancement of ROS generation compared to the combination of LPS and GN8 (Fig. 4D). These data indicated the presence of synergistic effects between several different MAMP elicitors.

## Discussion

In this paper, we showed that the pretreatment of rice cells with a very low concentration of LPS from several bacterial strains apparently primed the defense responses induced by successive chitin oligosaccharide treatment. These results seemed coincide well with the priming phenomena previously reported for LPS using intact plants and pathogen infection [8]. Ortmann *et al*. also reported the priming activity of EPS from *Pantoea agglomerans* on the ROS generation and peroxidase activity in several plant cells using similar model system [25], [26].

On the other hand, the fact that the simultaneous treatment of LPS and GN8 resulted in the similar enhancement of early cellular responses indicated that such a positive crosstalk of signaling cascade could be understood as a synergistic action of different MAMP molecules rather than priming. Aslam *et al*. also reported a synergistic action of two MAMP molecules, flg22 and LOS, in eliciting calcium influx, one of the earliest cellular responses induced by MAMPs [27], indicating the presence of positive crosstalk at an early step of signaling cascade. Our results further indicated that such a positive crosstalk of signaling cascade is not restricted to LPS and chitin oligosaccharides but also observed for other sets of MAMP molecules, suggesting that such a positive crosstalk of MAMP signaling may play an important role for effective mobilization of defense responses in plants.

Synergistic activation of immune responses by different MAMP molecules have also been observed for mammalian systems, especially for toll-like receptor (TLR)-mediated responses. Simultaneous addition of ligands for different TLRs synergistically activated downstream responses such as the production of interferons and interleukins [28]–[30]. It was suggested that multiple factors in the signaling cascade, such as NFκB, IRF, MAPK, PI-3K and STAT, are involved in the synergistic activation of cytokine genes [28]. On the other hand, synergistic effect of LPS and peptidoglycan on cytokine release was reported to be preceded by a reciprocal upregulation of TLR2 and TLR4, suggesting the receptor upregulation is involved in this process [31]. Similar mechanisms might be involved in the synergistic activation of defense responses observed in plants, though the evaluation of such possibilities remains for future studies.

**Figure 5:**
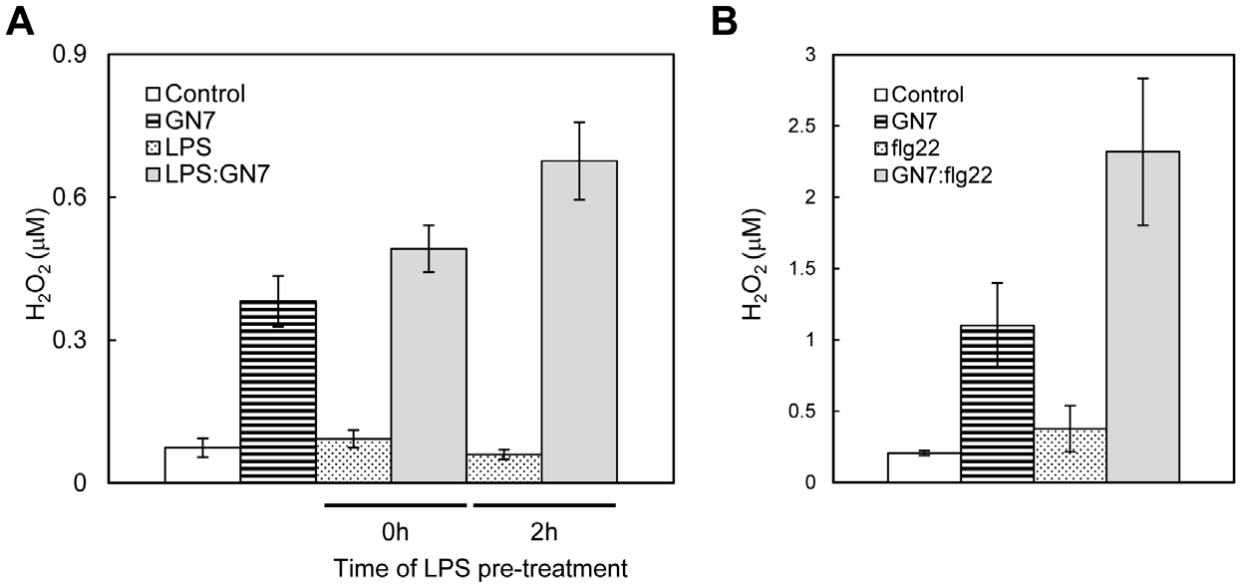
Synergistic effect on ROS generation in rice seedlings. (A) For priming experiments, LPS (5 µg/ml) was pretreated for 120 min before *N-Acetylechitoheptaose* (GN7, 10 ng/ml = 6.9 nM) treatment. In the case of simultaneous treatment, both LPS and GN7 were added simultaneously. The increment of ROS in 20 min after GN7 treatment was used as the index of defense response. (B) ROS generation induced by simultaneous treatment of GN7 (10 ng/ml) and flg22 (10 ng/ml = 4.4 nM).

The fact that the simultaneous treatment of LPS and GN8 resulted in a similar enhancement of defense responses observed for LPS pretreatment and successive GN8 treatment indicates that priming and synergy, which have been separately discussed so far, may have close relationships in some cases. Interestingly, when we tried similar experiments with rice seedlings instead of cultured cells, we observed that the simultaneous treatment of LPS and *N*-acetylchitoheptaose (GN7) showed a much less enhancement of ROS generation compared to the pretreatment of LPS and successive GN7 treatment (Fig. 5A). Thus, in the seedling assay, priming effect was clearly visible but synergistic effect was less clear for the combination of these MAMPs. One possible explanation for this discrepancy between the results obtained with these two experimental systems is the possible difference in the penetrating ability of these MAMP molecules in intact plant tissues. Because of its higher molecular weight, LPS is expected to penetrate more slowly than chitin oligosaccharides in the real plants where differentiated tissues form a tougher barrier compared to the cultured cells and reach the putative receptor on the cell surface much later than chitin oligosaccharides, making it difficult to observe as a synergistic action. On the other hand, synergistic enhancement of ROS generation was clearly observed for the combination of two low-molecular weight MAMP molecules, flg22 and GN7, even in the seedling assay (Fig. 5B). Thus, whether the crosstalk is recognized as priming or synergy seems to depend on the size and nature of MAMP molecules as well as the experimental systems, at least for the combination of these MAMP molecules and experimental systems similar to those described here. Interestingly, most synergistic actions of MAMP/DAMP molecules so far reported, especially for early responses, have been observed with cultured plant cells.

Considering the diversity of the factors proposed for priming/synergy so far, plants seem to have developed diverse ways of positive crosstalk in the defense signaling to prime and enhance defense responses, so that they can protect themselves more effectively from the invasion of pathogens.

## Materials and Methods

### Plant Materials

Suspension-cultured rice cells (*Oryza sativa* L. cv. Nipponnbare) were maintained using modified N-6 medium as described previously [32]. Rice cells were incubated on a rotary shaker at 25°C and 120 rpm in the dark and were transferred to fresh medium every week. After every other transfer to new medium, the cell clusters were filtered through a 20-mesh screen to generate fine aggregates, and then were used for the next culture. Cells harvested 4 or 5 days after transfer to the new medium were used in the experiments. For seedling assay, rice seeds were sterilized by sodium hypochrolite and spreaded on a petri dish (90 mm diameter) with 13 ml of water (fifteen seeds per dish). Germinating seeds incubated for 2 days at 30°C under light condition were used for the experiments.

### Elicitors

LPS preparations from *Pseudomonas aeruginosa* and *Escherichia coli* were purchased from Sigma Chemical Co. (St Louis, MO, USA, purified by gel filtration chromatography) and Wako Pure Chemical Co. (Osaka, Japan, obtained by phenol extraction), respectively. LPSs from *Xanthomonas oryzae pv. oryzae* and *Ralstonia solanacearum* were extracted by the hot-phenol method as described previously [7]. *N-Acetylchitoheptaose* and *N-acetylchitooctaose* were prepared by re-*N*-acetylation of the corresponding chitosan oligosaccharides, kindly supplied by Yaizu Suisan Kagaku Industrial Co. Ltd (Shizuoka, Japan). They were chosen rather arbitrarily for a specific experiment because both of these compounds showed comparable and enough eliciting activity for most experiments. Flg22 peptide was synthesized by Peptide Institute, Inc. (Osaka, Japan).

### Specific Adsorption of LPS by Polymyxin B–agarose Columns

Polymyxin B–agarose gel (Detoxi-GelTM) was purchased from Pierce Biotechnology Inc. (Rockford, IL, USA). A 300 µg aliquot of LPS was applied to a column containing 3 ml of Detoxi-GelTM and eluted with water. Flowthrough fraction (0.5 ml) was used for the experiments. A column packed with Sepharose CL-6B was used as a control column.

### Analysis of Reactive Oxygen Generation Induced by Elicitor Treatment

The induction of ROS in the medium of suspension-cultured rice cells by elicitor treatment was measured using the luminol assay [33] by the microtiter plate-based method [7]. Briefly, 40 mg of cultured cells were transferred to 1 ml of fresh medium in a 2 ml centrifuge tube and pre-incubated for 120 min at 25°C on a thermomixer shaken at 750 rpm. For priming experiments, cells were further incubated with LPS (0.1 µg/ml) for 120 min at 25°C, then the second elicitor, *N-Acetylchitooctaose* (0.08 ng/ml) was added. For the analysis of synergistic effect, cells were pre-incubated for 240 min, then both elicitors were added together. Ten µl aliquots of medium were then transferred to 96-well microtiter plates at various time points and immediately supplemented with 50 µl of 1.1 mM luminol and 100 µl of 14 mM potassium hexacyanoferrate solution using a programmable injector attached to the luminometer. Chemiluminescence was measured by a microplate luminometer model TR717 or centro LB960 (Berthold Technologies, Germany). The amount of ROS was estimated by using a standard curve for hydrogen peroxide. For seedling assay, each rice seedling was transferred to the well of a 48-well plate containing 500 µl of water and pre-incubated for 120 min at room temperature on a thermomixer shaken at 600 rpm. In the case of priming treatment, LPS (5 µg/ml) was added in the pre-incubation solution, then the elicitors (10 ng/ml) were added. Twenty µl aliquots of the incubation solution were used for ROS assay as described. As the background ROS level of each seedling was significantly different from seedling to seedling, the increment of ROS accumulation between 0 and 20 min was taken as the measure of elicitor response. Six seedlings were used for one treatment and the highest and lowest values were omitted.

### Analysis of Defense Gene Expression by Realtime–PCR

Rice cells were harvested 2 hours after GN8 added. Total RNA was extracted from rice cells using an RNeasy Plant Mini Kit (QIAGEN Inc., Valencia, CA, USA). The cDNA was amplified by QuantiTect Reverse transcription Kit (QIAGEN) and detected by Taq-man probes, using ABI 7500 Fast Real-time PCR System. The data was normalized to the amplification of 18S ribosomal RNA internal control. LPS concentration was 0.1 µg/ml and GN8 concentration was 0.08 ng/ml. The gene-specific primer pairs and probes were designed as follows:

*rBG*, forward primer, AGATGTCTATGCAAGGCGTTGTT, reverse primer, TGGATCGCATGCTGAAGGA, probe, CAGCGGCTTTGGCCATTGCA; *RCC1*, forward primer, GACATGTTGGGCGTCAGCTA, reverse primer, GCTAGAACGAGCTATTAGGAGTTGAAA, probe, CGCCAACTTGGACTGCTACAACCAGAG; *PAL*, forward primer, CGGTGTTGTTTTTATCTGGTGAAT, reverse primer, GAAACCTGCCACTCGTACCAA, probe, CATAGCGGCAAGCATGCAACAGCA; *OsDTC2*, forward primer, CCTTGTTCCCCTGCGAGTT, reverse primer, CATCAACCACAGCCGTCAGT, probe, CATTGCTTGGAGCCAGAACGCCA; *OsKSL4*, forward primer, TCGCATTGCGTGTGCAA, reverse primer, TTGGAACTTCCGACATCGAAA, probe, TCTATTGCGCTCACACTTGTTGCCGA; *18s rRNA*, forward primer, CAGATACCGTCCTAGTCTCAACCA, reverse primer, CGGCGGAGTCCTATAAGCAACAT, probe, AAACGATGCCGACCAGGGATCG.

### Comprehensive Analysis of Phytohormones

Sixty mg of rice cells were transferred to a 2 ml centrifuge tube containing 1.5 ml of fresh medium and pre-incubated for 120 min at 25°C on a thermomixer shaken at 750 rpm After LPS was added, the cells were incubated for 120 min at 25°C, then with the addition of *N-Acetylchitooctaose* for appropriate time. Phytohormones were extracted from 120 mg of rice cells and analyzed for JA, JA-Ile, IAA, ABA, SA, GA_1_, GA_4_, DHZ, tZ, iP and iPR. Methods of phytohormone extraction and analysis were described previously [34].

## Supporting Information

Figure S1 **Chitin-induced ROS generation was primed by various LPS preparations**. LPS preparations from *E. coli* (A), or phytopathogenic bacteria, *X. oryzae pv. oryzae* and *R. solanacearum* (B, C) were used for the experiments. Concentrations of LPS and GN8 were 0.1 mg/ml and 0.08 ng/ml, respectively.
(TIF)

Figure S2 **Changes in phytohormon concentrations after LPS pretreatment and successive GN8 treatment.** Rice cells were pretreated with LPS (0.1 mg/ml) 120 min before the successive GN8 (0.08 ng/ml) treatment. Auxin (indole-3-acetic acid), IAA; abscisic acid, ABA; salicylic acid, SA; gibberellin, GA_1_ and GA_4;_ dihydrozeatin, DHZ; trans-zeatin, tZ; isopentenyladenine, iP; isopentenyl adenosine, iPR. GA levels during the LPS pretreatment were below the detection limit.
(TIF)

Figure S3 **Changes in phytohormon concentrations after simultaneous treatment with LPS and GN8.** Rice cells were treated with LPS (0.1 mg/ml) and GN8 (0.08 ng/ml) simultaneously. Auxin (indole-3-acetic acid), IAA; abscisic acid, ABA; salicylic acid, SA; gibberellin, GA_1_ and GA_4;_ dihydrozeatin, DHZ; trans-zeatin, tZ; isopentenyladenine, iP; isopentenyl adenosine, iPR. GA levels during the LPS pretreatment were below the detection limit.
(TIF)
