# Supplementary figures and images for: Positive Crosstalk of MAMP Signaling Pathways in Rice Cells
Source: PLoS One. 2012 Dec 14;7(12):e51953. doi: 10.1371/journal.pone.0051953 (PMC3522599; doi:10.1371/journal.pone.0051953)

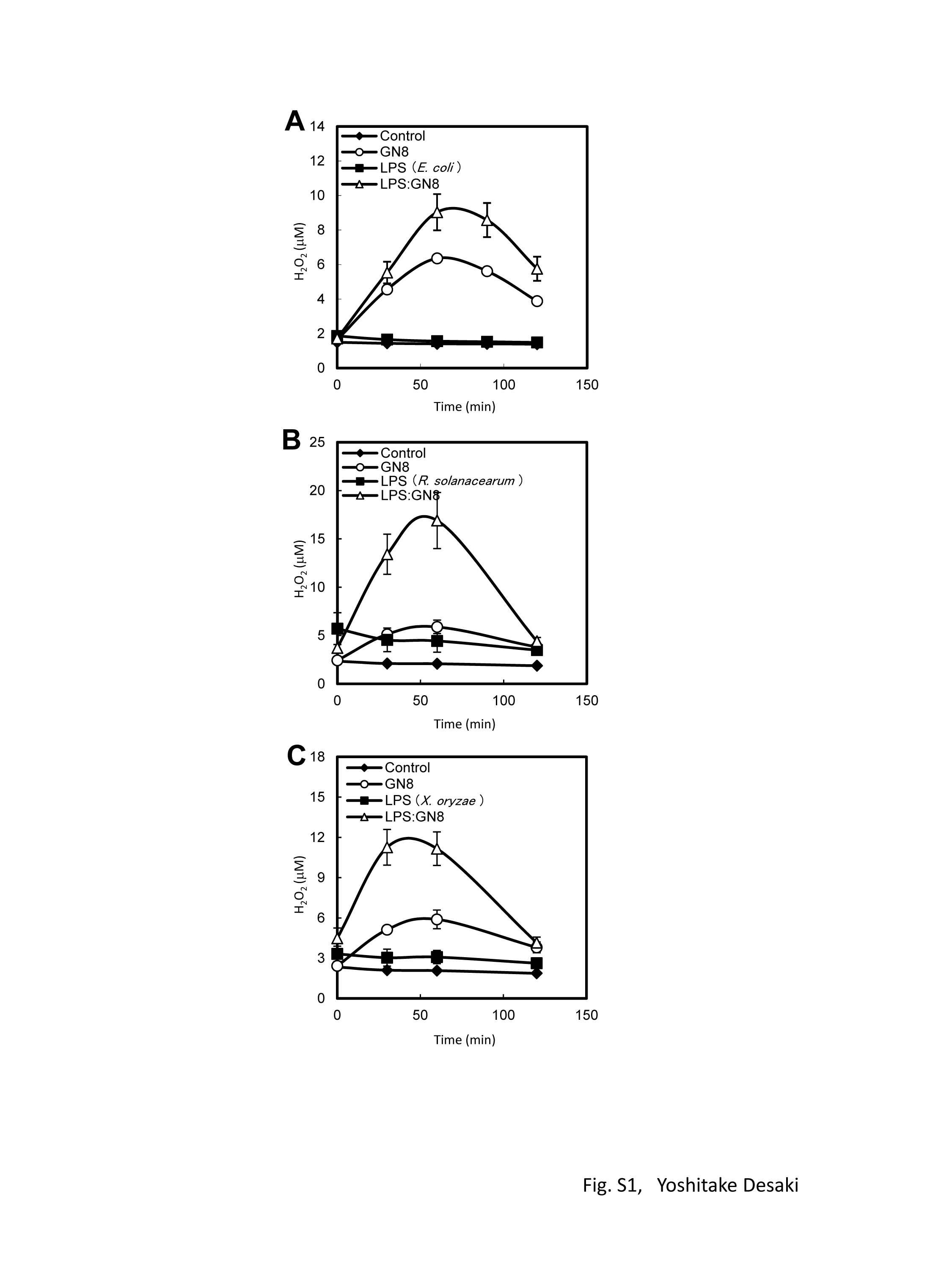

Supplement: Figure S1 — Chitin-induced ROS generation was primed by various LPS preparations. LPS preparations from E. coli (A), or phytopathogenic bacteria, X. oryzae pv. oryzae and R. solanacearum (B, C) were used for the experiments. Concentrations of LPS and GN8 were 0.1 mg/ml and 0.08 ng/ml, respectively. (TIF) [file pone.0051953.s001.tif]

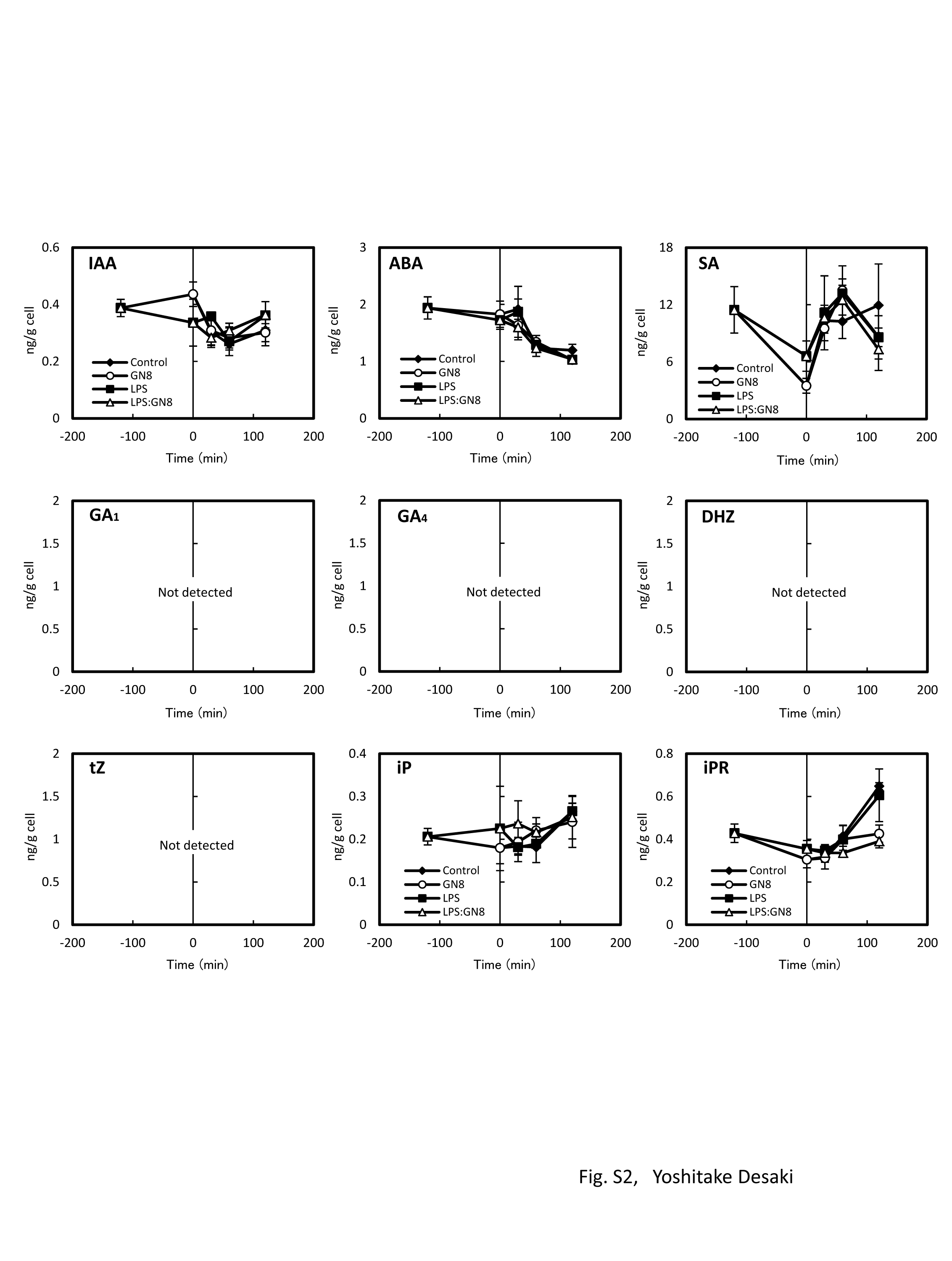

Supplement: Figure S2 — Changes in phytohormon concentrations after LPS pretreatment and successive GN8 treatment. Rice cells were pretreated with LPS (0.1 mg/ml) 120 min before the successive GN8 (0.08 ng/ml) treatment. Auxin (indole-3-acetic acid), IAA; abscisic acid, ABA; salicylic acid, SA; gibberellin, GA1 and GA4; dihydrozeatin, DHZ; trans-zeatin, tZ; isopentenyladenine, iP; isopentenyl adenosine, iPR. GA levels during the LPS pretreatment were below the detection limit. (TIF) [file pone.0051953.s002.tif]

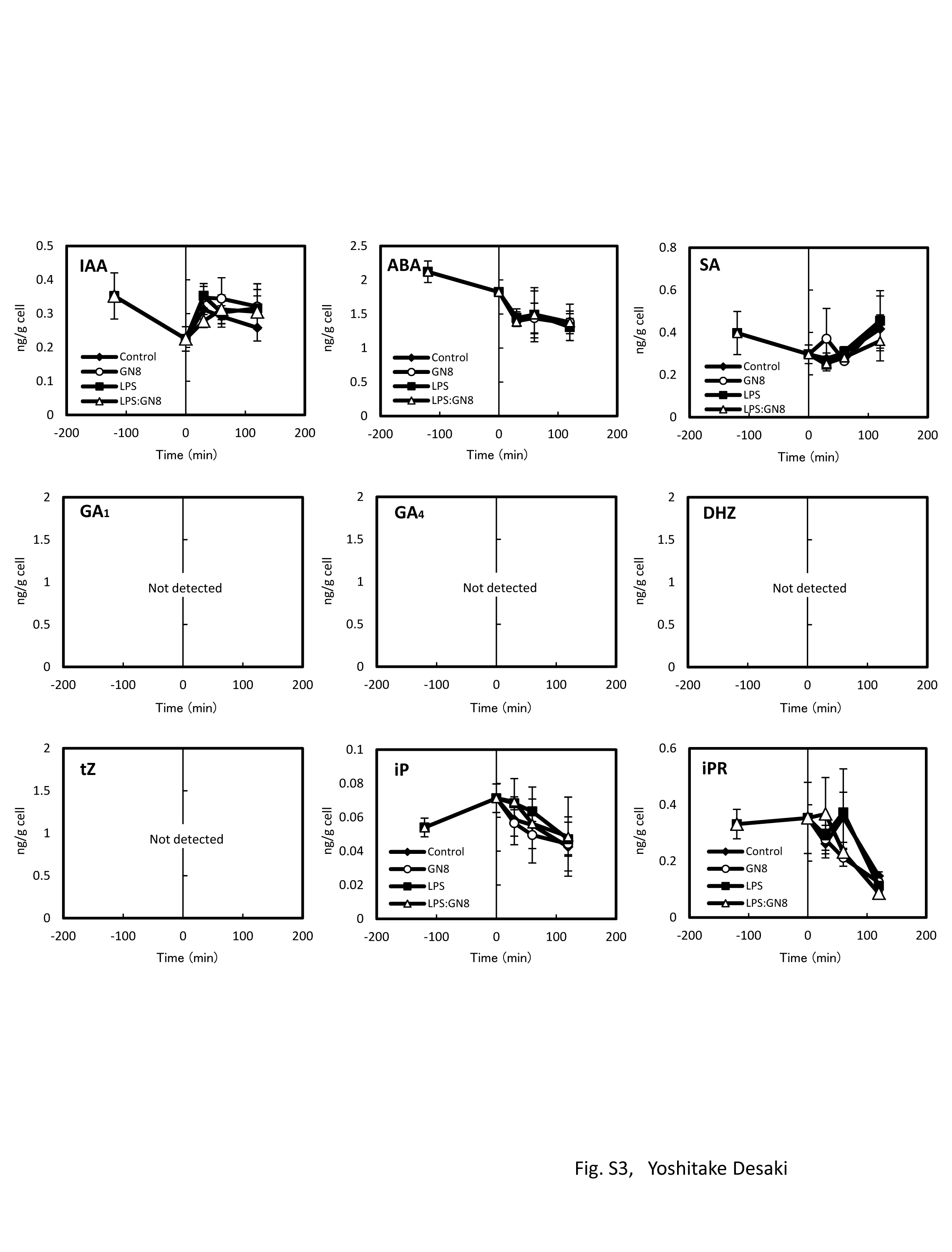

Supplement: Figure S3 — Changes in phytohormon concentrations after simultaneous treatment with LPS and GN8. Rice cells were treated with LPS (0.1 mg/ml) and GN8 (0.08 ng/ml) simultaneously. Auxin (indole-3-acetic acid), IAA; abscisic acid, ABA; salicylic acid, SA; gibberellin, GA1 and GA4; dihydrozeatin, DHZ; trans-zeatin, tZ; isopentenyladenine, iP; isopentenyl adenosine, iPR. GA levels during the LPS pretreatment were below the detection limit. (TIF) [file pone.0051953.s003.tif]
